# Scanning the Immunopathogenesis of Psoriasis

**DOI:** 10.3390/ijms19010179

**Published:** 2018-01-08

**Authors:** Andrea Chiricozzi, Paolo Romanelli, Elisabetta Volpe, Giovanna Borsellino, Marco Romanelli

**Affiliations:** 1 Dermatology Department, University of Pisa, Via Roma 67, 56126 Pisa, Italy; romanellimarco60@gmail.com; 2 Department of Dermatology and Cutaneous Surgery, University of Miami Miller School of Medicine, 1295 NW 14th St, Miami, FL 33125, USA; promanelli@med.miami.edu; 3 The Laboratory of Neuroimmunology, Fondazione Santa Lucia, Via del Fosso di Fiorano, 64, 00143 Rome, Italy; e.volpe@hsantalucia.it (E.V.); g.borsellino@hsantalucia.it (G.B.)

**Keywords:** psoriasis, pathogenesis, immunology, autoantigen, IL-17, IL-23, cytokines, chemokines, autoreactive T cells, dendritic cells

## Abstract

Psoriasis is a chronic inflammatory skin disease, the immunologic model of which has been profoundly revised following recent advances in the understanding of its pathophysiology. In the current model, a crosstalk between keratinocytes, neutrophils, mast cells, T cells, and dendritic cells is thought to create inflammatory and pro-proliferative circuits mediated by chemokines and cytokines. Various triggers, including recently identified autoantigens, Toll-like receptor agonists, chemerin, and thymic stromal lymphopoietin may activate the pathogenic cascade resulting in enhanced production of pro-inflammatory and proliferation-inducing mediators such as interleukin (IL)-17, tumor necrosis factor (TNF)-α, IL-23, IL-22, interferon (IFN)-α, and IFN-γ by immune cells. Among these key cytokines lie therapeutic targets for currently approved antipsoriatic therapies. This review aims to provide a comprehensive overview on the immune-mediated mechanisms characterizing the current pathogenic model of psoriasis.

## 1. Introduction

Plaque-type psoriasis is a chronic inflammatory skin disease involving both the innate and the adaptive immune compartments, crosstalking with skin tissue cells. 

The interaction between hyperproliferative keratinocytes (KCs), inflammatory dendritic cells (DCs), neutrophils, mast cells, and T cells, induces to the development of psoriatic lesions, clinically characterized by sharply demarked, erythematous, and scaly plaques. In the last three decades, the pathogenic model for psoriasis has been profoundly revised according to a broader and deeper understanding of the immune mechanisms leading to plaque formation. 

**Figure 1:**
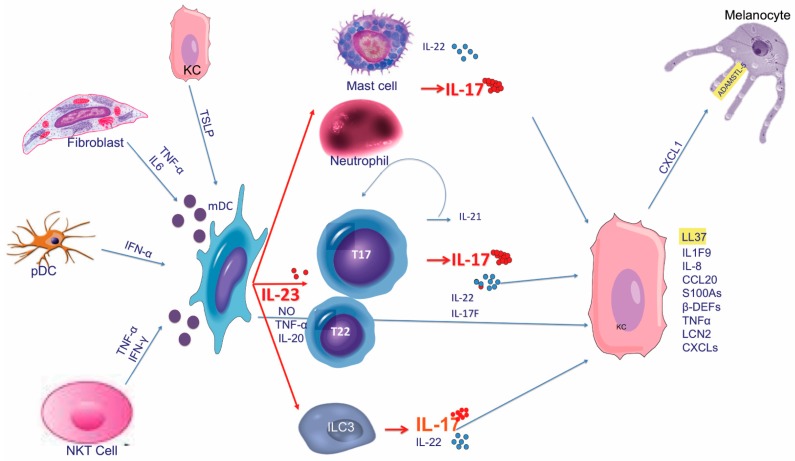
The pathogenic model based on the IL-23/IL-17 axis inducing the development of psoriatic phenotype. Multiple factors induce mDCs activation with consequent IL-23 production (IFN-α, TSLP), that, in turn, stimulates mainly T cell subsets, but also ILC3, mast cells, and neutrophils, to secrete IL-17. Other cytokines derived from T cells, mast cells, and ILC3 (IL-22, IL-17F, and IL-21), and from mDCs (TNFα, NO, and IL-20) contribute to the development of psoriatic skin. Autoantigens involved in this pathway are highlighted in yellow. T17 and T22 cells represent all T cell subsets producing mainly IL-17 and IL-22, respectively. The IL-23/IL17 axis, the main immune pathway in psoriasis pathogenesis, is highlighted in red, while the other immune signals are designed in blue. CCL: CC chemokine ligands; CXCL: chemokine (C-X-C motif) ligand; ADAMTSL5: Thrombospondin Type 1 motif-like 5; β-DEF: β-defenins; IFN: interferon; IL: interleukin; KC: keratinocyte; mDC: myeloid Dendritic Cell; NKT: Natural Killer T cell; NO: nitric oxide; pDC: plasmacytoid Dendritic Cells; TNF: tumor necrosis factor; TSLP: Thymic stromal lymphopoietin.

Before the late 1990s, there was a debate on whether KC proliferation was due to intrinsic KC defects triggering an immune response or, viceversa, whether KC hyperproliferation was a secondary phenomenon induced by immune activation and inflammation. In 1995, a milestone study demonstrated psoriatic plaque resolution following selective apoptosis of activated T cells, without affecting KC survival or activation, thus demonstrating the crucial role of the immune system, particularly of T cells, in the disease [1]. This immune hypothesis found confirmation in numerous studies and effective immune-targeting therapies [2,3,4,5]. Initially, the pathogenic mechanism was thought to be based on the upregulation of interferon (IFN)-γ and interleukin (IL)-12, signaling, with both cluster of differentiation (CD)4+ and CD8+ IFN-γ-producing T cells (named Th1 and Tc1 cells, respectively) as key players [6,7]. More recently, an accurate characterization of the immune pathways involved in psoriasis led to recognize the role of specific subsets of immune cells and their derived products with the subsequent identification of new therapeutic targets. Thereby, the pathogenic paradigm has been profoundly revised in favor of an IL-23/IL-17 axis (Figure 1) [8,9,10]. IL-23 is the most potent inducer of IL-17 production by different cell types, including T cells (named in this manuscript T17 cells), neutrophils, ILC3, NK, NKT cells, and mast cells, that have all been recognized as strong contributors to the pathogenesis in psoriasis [11]. Gene products involved in the psoriatic inflammation represent a consistent part of the genetic susceptibility that has been progressively established by numerous GWAS within the last ten years. The identification of psoriasis risk genetic loci, so-called PSORS 1-9, has been improved by GWAS that more accurately described specific susceptibility genes associated with psoriasis, giving reason of its peculiar immunologic profile [12,13,14,15,16,17,18,19,20,21,22]. Indeed, these studies led to a better understanding of the pathogenic hierarchy and relevance of certain cell types, intracellular signaling pathways, or mediators (cytokines, chemokines, antimicrobial peptides, etc.) in psoriasis compared to other inflammatory skin disorders, wherein the involvement of the same cell types, cytokines, or signaling pathways determine different pathogenic effects. 

More recently, the clinical interest in psoriasis-related comorbid conditions has fueled the investigation on immune-mediated circuits or mediators that could link psoriasis to its comorbidities. The view of psoriasis shifted from a mere skin disease to a skin disorder associated to systemic inflammation and a wide array of concomitant conditions, mirroring other tissue-specific diseases with systemic implications such as Crohn’s disease, and rheumatoid arthritis (the so-called immune-mediated inflammatory diseases, IMIDs) [23]. Hence, psoriatic skin inflammation is not confined to the lesional site, as high levels of cytokines and activated immune cells circulate into the bloodstream affecting also distant uninvolved skin, and other tissues or organs [24,25,26,27,28,29,30,31]. This review aims to illustrate the immune pathogenic mechanisms in psoriasis, with a focus on the cellular and soluble contributors, and a survey of the current pathogenic model.

## 2. Main Cell Types Involved in Psoriasis

A large plethora of immune cells contribute, to different extents, to the pathogenesis of psoriasis. In this section, we will illustrate the role and the most relevant supporting evidence of each cell type.

### 2.1. T Cells

#### 2.1.1. T Helper and Cytotoxic T Cells

The role of T cells in the pathogenesis of psoriasis has been well described, and both CD4+ T cells (T helper cells, Th) and CD8+ T cells (cytotoxic T cells, Tc) seem to be critical in the development of the skin lesions [27,31,32,33,34,35]. The injection of CD4+, and not CD8+, T cells obtained from psoriatic patients into human non-lesional skin in vitro, and then grafted onto immunodeficient mice model (SCID mice), has been shown to be responsible for psoriasis development [36]. This CD4+ T cell-driven process is then followed by CD8+ T cell activation and recruitment. On the other hand, the development of psoriatic-like skin in a mouse model is inhibited by CD8+, and not CD4+, T cell depletion [37]. Conversely to the CD4+ T cell-based psoriasis model, an early epidermal infiltration of CD8+ T cells is thought to be essential for the onset of psoriasis inflammation, rather than the dermal infiltration of CD4+ T cells [38,39]. Moreover, the primary role of CD8+ T cells is underlined by the identification of human leukocyte antigen (HLA)-C*06:02 as susceptibility gene, a HLA class I molecule presenting peptide antigens to CD8+ T cells, not CD4+ T cells [40]. 

Overall, in human lesional skin as well as in the bloodstream the number of both CD4+ and CD8+ T cells is increased [27,31,32,34,35]. These cells express CLA and chemokine receptors, and penetrate in the skin interacting with endothelial cells expressing adhesion molecules, such as P-selectin and E-selectin. This gives reason of the marked infiltration of CD4+ and CD8+ T cells in the dermis and epidermis of lesional psoriatic skin, respectively [27,31,32,34,36]. 

Based on their cytokine production, multiple subsets of CD4+ lymphocytes (Th) have been identified within the cellular infiltrates: Th1, Th17, Th9, follicular Th, and Th22 cells, as have their CD8+ counterparts (Tc). Specifically, Th1 and Tc1 peculiarly show (i) signal transducer and activator of transcription 1 (STAT1) and T-bet expression as signature transcriptional factors [41]; (ii) release of IFN-γ, TNF-α, and IL-2; (iii) expression of the CXCR3 as chemokine receptor; and (iv) differentiation driven by IL-12 [6,7,32,42,43,44,45]. Th17 and Tc17 (i) express STAT3 and RORγt as signature transcriptional factors; (ii) release IL-17, IL-17F, TNF-α, IL-21, IL-22, and IL-26; (iii) express IL-23 receptor, the chemokine receptors CCR6 and CCR4 [46,47]; and (iv) differentiate in presence of IL-23, IL-1β, TGF-β, and IL-6 [48,49]. Th22 and Tc22 (i) express STAT3 expression as signature transcriptional factor; (ii) release IL-22; (iii) bear CCR10, CCR6 and CCR4, as chemokine receptors; and (iv) their differentiation is driven by TNF-α and IL-6 [50,51]. Other Th cell subpopulations, such as Th9 and Follicular Th cells, have been reported to contribute to the pathogenesis of psoriasis through the enhancement of the most relevant immune pathways, in particular the IL-17 signaling [52,53]. 

#### 2.1.2. γδ T Cells

Recent studies have revealed that the majority of IL-17-producing T cells in both human and murine psoriasis express the γδ T cell receptor [54,55]. These cells produce IL-17 and IL-22 upon stimulation with IL-23 or IL-1β, and they share multiple features with other IL-17-producing cells (i.e., Th17 and Tc17 cells): they constitutively express the IL-23 receptor, CLA, skin homing chemokine receptors (i.e., CCR6), and the transcription factor RORγt [54,55]. Upon stimulation with IL-23 or IL-1β, they are able to produce IL-17 and IL-22. IL-23 stimulation also induced dermal and epidermal infiltration, as described in two distinct psoriasis mice models [56]. Similarly to IL-17 receptor-deficient mice model, T cell receptor γδ-deficient mice showed significant reduction of psoriasiform pathologic features, after challenge with recombinant IL-23 or imiquimod [56]. Moreover, in human lesional psoriatic skin, a marked infiltration of IL-17-producing γδ+ T cells was detected with an absolute cell number resulting significantly higher than IL-17-producing γδ- T cells [56]. 

### 2.2. Dendritic Cells

Various subtypes of DCs can be detected in normal and pathological skin [57]. However, only two subtypes, namely pDCs and inflammatory mDCs, seem to profoundly contribute to psoriasis pathogenesis. They act as potent antigen presenting cells but also as relevant sources of key pathogenic mediators including TNF-α and IL-23. On the contrary, the pathogenic role of epidermal Langerhans cells (LCs) is still uncertain.

#### 2.2.1. Plasmacytoid DCs

pDCs are identified by the phenotype HLA-DR+CD11c-CD123hiBDCA-2+ [57]. These cells produce large amounts of type 1 interferons (IFN-α, β, ω) during viral infection, following the bind of single strand RNA or DNA to endosomal Toll-like receptor (TLR)7 and TLR9, respectively [58,59], and they are considered the primary source of IFN-α in the skin. Their activation, leading to abundant IFN-α production, represents one of the *primum movens* in psoriasis pathogenesis: first, IFN-α regulates the development and maturation of T cells and myeloid DCs, that markedly express the IFN receptor [60]; second, it triggers a downstream mechanism leading to the development of the psoriatic phenotype. Activating pDCs through TLR7, imiquimod application was able to induce the psoriatic phenotype in human subjects as well as in mice models [61,62]. In these models, an increased pDC-derived IFN-α production was found, mirroring the enriched infiltration of pDCs and the greater expression of IFN-α detected in human lesional as compared to non-lesional psoriatic skin [61,62,63]. Their recruitment is induced by various chemoattractans as they bear multiple chemotactic receptors, including CXCR4, CXCR3, CCR5, and ChemR23 (chemerin receptor) [64,65,66,67,68,69]. Besides imiquimod, pDCs could be activated by various triggers including chemerin and other TLRs agonists: DNA or RNA deriving from damaged cells and complexed with LL37, β-defensins, lysozyme, or IL-26 [70,71,72,73]. pDC cell activation is crucial in psoriasis pathogenesis as proven by a murine model of psoriasis wherein the development of skin lesions is inhibited by anti-BCDA-2 antibody, which suppresses pDC activation and, thus, IFN-α production [63].

#### 2.2.2. Myeloid DCs

The mDCs subpopulations, characterized by the positivity for CD11c, are abundant in the lesional psoriatic skin. These cells are thought to derive from circulating precursors that migrate into the skin because of inflammatory and chemotactic signals, and differentiate in the psoriatic inflammatory milieu [74,75,76,77,78,79].

Two mDC subpopulations can be distinguished:

(i) CD11c+CD1c- cells, which are phenotypically immature, produce inflammatory cytokines (TNF and IL-6), and represent the most prevalent CD11c+ subpopulation infiltrating psoriatic skin [80,81,82,83]. These relatively immature mDCs, also known as Tip-DCs or inflammatory mDCs, are considered crucial players in psoriasis pathogenesis [57]. Indeed, they secrete TNF-α, IL-6, IL-20, IL-23 (and IL-12), they express iNOS, producing NO [79,80,81,82,83,84]. Because of this activity, they are able to induce inflammation (through TNF-α and NO), epidermal hyperplasia (through IL-20), and T cell differentiation (through IL-12 and IL-23) [80,81,82,83]. Although mDCs are able to secrete both p40 cytokines, IL-12 and IL-23, that consequently drive T cell differentiation towards a Th/Tc1 and Th/Tc17 phenotype, they mostly release IL-23 that sustains and amplifies the IL-17-mediated response, whereas IL-12 expression is not upregulated in lesional skin compared to non-lesional skin [80,81,82,83]. Dermal Tip-DC infiltration detected in lesional psoriatic skin is estimated as 30-fold greater than normal skin and 10-fold greater than non-lesional psoriatic skin [57,84,85].
(ii) A second population of mDC characterized by the phenotype CD11c+ DC-LAMP+ DEC-205/CD205+BDCA-1+, acts as resident mature antigen-presenting cell and is phenotypically similar to those contained in normal skin. The number of these DCs does not increase in lesional skin compared to uninvolved skin [57,82]. These mature “resident” DCs are likely responsible for the antigen presentation to cutaneous T cells occurring in situ [86], within the dermis rather than following migration to draining lymph nodes [82,87]. CD1c+ “resident” DCs, representing mature (DC-LAMP/CD208+, CD205+, and CD86+) DCs, establish dermal clumps with T cells constituting lymphoid tissue-like structures [80,81,82,83,86,87], though T cells can be stimulated by Tip-DCs (CD11c+, CD1c- mDCs) as well [57]. Therefore, beyond the classic role of antigen-presenting cells, Tip-DCs show a prominent inflammatory activity in psoriasis and their infiltration is increased in lesional skin but normalized during treatment with effective therapies [85,88].

### 2.3. Neutrophils

Neutrophils infiltrate the dermis in the early phase of the psoriatic plaque formation, and subsequently they migrate into the epidermis, aggregating in microabscesses (Munro’s microabscesses), which represent one of the histopathological features of the disease. The ligands for CXCR2, such as CXCL-1, CXCL-2, CXCL-8 (also known as IL-8), and antimicrobial peptides (AMPs), are abundantly expressed in lesional psoriatic skin [89], mainly produced by KCs upon IL-17, IL-22, and TNF stimulation [90,91,92,93,94]. Neutrophils constitute a relevant source of pro-inflammatory mediators, including IL-17 that is, at the same time, a factor inducing their survival, recruitment, and activation [95,96]. Since they express the IL-17 receptor, IL-17 could constitute an important autocrine autoamplifying signal [97]. The presence of IL-17 embedded into cytoplasmic vesicles has been described, whereas it is still debated whether neutrophils are able to express mRNA codifying for IL-17 [95,96,97,98,99,100,101,102,103]. Some studies hinted to neutrophils as relevant sources of IL-17 that is released through extracellular traps and conventional degranulation through their expression of RORγt, whose activation is regulated by IL-23 and IL-6 [95,97]. In vivo models of human skin inflammation that share many histological features with psoriasis revealed an enhanced expression of both IL-17 and the IL-17-associated transcription factor RORγt in neutrophils, and the majority of IL-17 was expressed by both neutrophils and mast cells, and not by T cells [95,97,101,103]. 

Although in certain reports IL-17+ neutrophils have been found to pronouncedly infiltrate lesional psoriatic skin, some authors reported low or undetectable IL-17 mRNA expression by neutrophils [98,99,103]. Since IL-17 mRNA is undetectable in neutrophils, it has been hypothesized that they are a reservoir for IL-17 produced by other cells, internalized and stored in the cytoplasm, and released extracellularly upon activation through the extracellular trap formation [87,95,101]. Moreover, neutrophils do not respond to IL-23 only, but also to IL-6, and IL-17, thus their IL-17 expression and secretion could be not strictly dependent on IL-23 stimulation, as observed in palmo-plantar pustolosis and palmo-plantar pustular psoriatic skin, wherein the high number of IL-17+ neutrophils in lesional skin is counterpointed by a scattered infiltration of IL-23+ mDCs [104].

### 2.4. Mast Cells

Mast cells belonging to the innate immune compartment and are known to infiltrate lesional skin during the early phases of psoriatic plaque formation [105,106,107,108,109]. They produce pro-inflammatory factors including IL-8, IL-22, and IL-17 [107,108]. Evidence of a high number of mast cells involved in the early steps of the pathogenic cascade and their ability to produce key pathogenic mediators [107,108] has been reported in a seminal study by Girolomoni’s group, where mast cell infiltration was associated with the presence of pDCs and neutrophils within the dermis, and with mast cell-derived chemerin production [109]. A recent study also revealed their capability (i) to express mRNA transcripts codifying for both IL-22 and IL-17; and (ii) to release cytokines through the formation of extracellular traps or degranulation, as occurs for IL-17, upon stimulation with IL-23 and IL-1β [95,108]. In particular, mast cells have been reported to be the major IL-22-producing cell type in lesional skin, while IL-17 is mostly derived from T cells and only a relatively small portion can be attributed to mast cells [108]. On the contrary, another study reported mast cells to be one of the predominant producers of IL-17 in psoriatic lesional skin as well as in normal skin [95]. 

### 2.5. NK Cells and NK-T Cells

Natural killer (NK) cells, CD56+CD16+ cells, and NKT cells (which share features from both T cells and NK cells) constitute a heterogeneous subset of immune cells that are significantly increased in psoriatic lesional skin and that are likely implicated in psoriasis pathogenesis [110,111]. Similar to pathogenic T cell subsets, these cells have the ability of producing pathogenic cytokines, such as IFN-γ, IL-17, TNF-α, and IL-22 and, particularly NKT cells, express chemokine receptors, such as CXCR3, CCR5, and CCR6, that facilitate their recruitment in lesional skin [112,113,114]. Although it is clear that these cells may contribute to inflammation, as indicated by the development of psoriasis driven by activated NKT cells in mice models grafted with normal skin or non-lesional skin, their function and their pathogenic role are not fully understood yet [113,114]. 

### 2.6. Innate Lymphoid Cells

Innate lymphoid cells (ILCs) represent a heterogeneous group of immune cells lacking specific antigen receptors or T/B cell markers. They are thought to be crucially involved in tissue remodeling, tissue protection, and lymph node formation during fetal development [115]. Among ILC subsets expressing NKp44 are the ILC3, which express the transcription factor RORγt and upon stimulation with IL-1β and IL-23 produce both IL-17 and IL-22 and are thought to be involved in the pathogenesis of psoriasis [116,117,118]. The number of NKp44+ ILC3 is significantly higher in lesional skin compared to non-lesional psoriatic skin [116], and is consistently higher in the bloodstream of psoriatic patients compared to healthy individuals or atopic dermatitis patients [116]. Moreover, a reduction of infiltrating and circulating ILC3 is observed during anti-TNFα therapy [118].

Although some authors suggest that this ILC subset may be considered a good biomarker of disease activity and a relevant contributor of the disease, its pathogenic role still needs to be clarified.

### 2.7. Keratinocytes

Since keratinocytes bear receptors for the majority of psoriasis-signature cytokines, they represent the “key responding” tissue cells to the psoriatic microenvironment. They respond to psoriatic cytokines by proliferating and amplifying inflammation through the production of other cytokines (i.e., IL-1F9, (IL-36γ), TNFα, IL-17C, IL-19, TSLP), chemokines (i.e., CCL20, CXCL1, CXCL8-11), proliferation-stimulating factors (EGF, VEGF, and HBEGF), and other pro-inflammatory products, such as AMPs [90,92,119,120,121,122]. Specifically, each cytokine modulates distinct keratinocyte-response pathways with a certain degree of overlap in their gene expression induction [90,92,94,119,122,123]. For instance, IL-17 and TNF-α strongly induce the synthesis of pro-inflammatory mediators with additive and synergistic effects on keratinocyte gene expression [90,123]; similarly, IL-22 and other IL-20 cytokine family members (i.e., IL-19 and IL-20) stimulate keratinocyte hyperplasia, promoting epidermal thickness [124,125,126]. Once activated, keratinocytes participate to pathogenic circuits that sustain and amplify skin inflammation releasing chemokines and other chemoattractants (i.e., CCL20, CXCL1, CXCL8-11, antimicrobial proteins), which are crucial for the recruitment of T cells, neutrophils, and inflammatory myeloid dendritic cells. Although keratinocytes have a relevant role in mediating inflammation, this hypothesis considers keratinocyte response as secondary to immune cell activation. However, a recent study confirmed their immune relevance showing that keratinocyte genetic defects yield mice more susceptible to specific IL-17-mediated psoriasis-like inflammation. For instance, keratinocytes lacking Tnip1, a psoriasis susceptibility gene codifying for a negative regulator protein involved in various inflammatory signaling pathways, including TNF receptor and TLRs pathways, show psoriasis-like inflammation associated with upregulation of IL-17 signaling upon application of low-dose imiquimod [127].

## 3. Main Cytokines in Psoriasis

**Figure 2:**
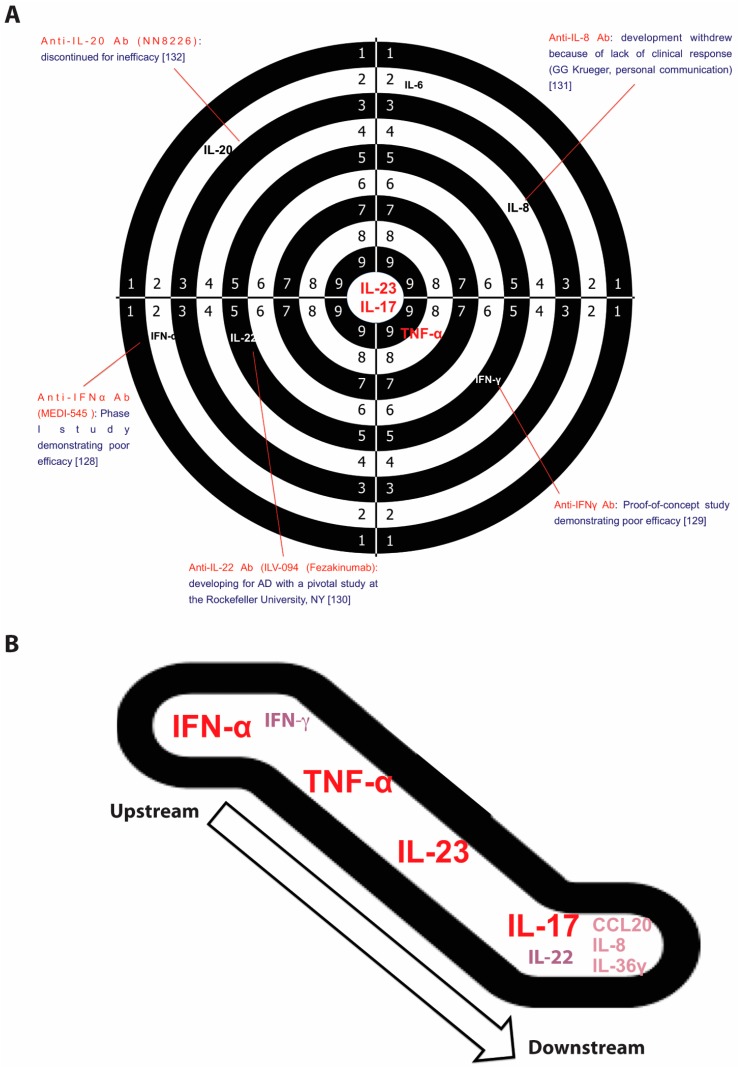
Therapeutic “hierarchy” of pathogenic cytokines in psoriasis. (**A**) The shooting target shows the best targets for treatment of psoriasis (IL-17, IL-23, and TNF-α). Moving away from the center, other pathogenic cytokines have proved to be less therapeutically relevant because their blockade resulted in a poor clinical response [11,128,129,130,131,132]; (**B**) key-cytokines (IFNα, TNFα, IL-23, and IL-17) in upstream and downstream points within the psoriatic inflammatory cascade, and other relevant contributors: IFN-γ, IL-22, IL-1F9, IL-8, and CCL20. CCL: CC chemokine ligands; IFN: interferon; IL: interleukin; TNF: tumor necrosis factor.

Pathogenic circuits involve multiple mediators, including cytokines that are currently identified as the most druggable targets. Functional studies in animal models, in vitro experiments, transcriptomic and ex vivo evidence, successful (and unsuccessful) clinical experiences in treating psoriasis have all helped define the role of each cytokine in inducing the psoriasis phenotype and its therapeutic relevance (Figure 2A). 

### 3.1. Interferon (IFN)-α

IFN-α belongs to the type I interferon family that also includes IFN-β, -κ, -δ, -ε, -τ, -ω, and -ζ. It is produced by pDCs and, similar to other type I IFNs, it strongly activates immature mDCs to produce IL-12, IL-15, IL-18, and IL-23 [71]. IFN-α is considered to be one of the initiators of psoriasis inflammation acting as an upstream cytokine along the IL-23/IL-17 axis (Figure 2B). Its role was initially suggested by the exacerbation of psoriatic lesions or by new-onset psoriasis following IFN-α therapy for viral infections [133,134,135]. A similar clinical behavior was also described using imiquimod, a TLR7 agonist inducing type I IFN production by pDCs [61]. Furthermore, IFN-α-induced genes are upregulated in lesional psoriatic skin, compared to non-lesional and normal skin. Another evidence supporting the role of IFN-α in psoriasis derives from a study showing that IFN-α neutralization prevents the spontaneous development of psoriatic lesions in mice xenotransplanted with non-lesional skin obtained from psoriasis patients [63]. In this model the development of psoriatic lesions was associated with an increase of IFN-α levels, demonstrating its pathogenic role [63]. Moreover, another mice model lacking a transcriptional factor, IRF-2 (IFN regulatory factor-2), which belongs to the of IFN-α/β pathway and acts as downregulating factor, spontaneously developed new psoriasiform skin lesions, characterized by CD8+ infiltrating T cells and increased expression of type I IFN-inducible genes [136]. However, a clinical trial (phase I) testing MEDI-545, an anti-IFN-α agent, in patients with plaque-type psoriasis did not show clinical improvement [128].

### 3.2. Interferon (IFN)-γ

Prior to the revolutionizing “IL-17-centric” pathogenic model, the IL-12/IFN-γ axis was considered to be essential in the pathogenesis of psoriasis. Given the profound revision of the pathogenic mechanisms of this disease, also the IL-12/IFN-γ axis role needs to be re-defined. High IFN-γ expression levels were detected in lesional skin, uninvolved skin, and in serum. In particular, levels of IFN-γ in serum and lesional skin correlate with disease severity. However, successful therapies dampen Th1 cells and Tc1 infiltration, although the clinical response does not correlate with the suppression of IFN-γ but rather with IL-17 signaling [84,137]. 

In ex vivo lesional psoriatic skin, IFN-γ upregulates the expression of approximately 400 genes, through the activation of signal transducer and activator of transcription 1 (STAT1)*,* an IFN-γ-signature transcription factor [119,120]. In vitro, IFN-γ stimulation alters the expression of approximately 1200 genes in monolayer keratinocytes [92]. Notwithstanding the large set of genes regulated by IFN-γ, in a 3-D skin model the set of genes regulated by IFN-γ results weakly enriched in the psoriasis transcriptome compared to that regulated by IL-17 [119]. The hypothesis that considers IFN-γ as an IL-17 suppressor has been revised in light of the recent findings showing co-production of both IL-17 and IFN-γ by Th17 cells, in particular if stimulated in vitro with IL-12 [137,138]. This subset of IFN-γ/IL-17-producing T cells has been detected in psoriatic lesions as well as in allergic contact dermatitis [139]. In a murine model of diabetes, it has been demonstrated that IL-17-producing cells become more pathogenic when they also produce IFN-γ [140,141]. This evidence is in line with previous studies demonstrating the ability of both Th1 and Th2 cells to produce IL-17 [142]. Moreover, IFN-γ may also play a role as an upstream cytokine in the IL-23/IL-17 axis, driving production of IL-23 and IL-1β by mDC and promoting IL-17 production by memory T cells [143,144]. The ability of IFN-γ to promote inflammation in psoriasis was underlined by a seminal study demonstrating that a single intradermal injection of IFN-γ in clinically unaffected skin of both psoriasis patients with mild disease (<10% BSA) and healthy volunteers, induces a transcriptomic signature and cellular infiltration pattern, similar to lesional psoriatic skin [138]. The transcriptomic analysis of IFN-γ-treated psoriatic skin showed upregulation of 775 unique differentially expressed genes (DEGs) and downregulation of 719 DEGs (708 probe-sets); however, no significant differences were found in comparison to IFN-γ-treated skin from healthy volunteers [138]. Among the upregulated genes, inflammatory mediators typical of psoriasis, including TNF-α, iNOS, IL-23p19, CCL19, ICAM-1, VCAM-1, and TRAIL were detected, concomitantly with an increased dermal infiltration of CD3+ T cells and CD11c+ DCs [138]. These observations are in line with previous studies reporting the development of psoriatic skin lesions after IFN-γ injections [145], and the downregulation of DC-derived products, including IL-23p19, IL12/23p40, and iNOS by therapeutic IFN-γ-neutralization, confirming IFN-γ regulation on DC activity [129]. Moreover, the potential role of IFN-γ in the early pathogenic steps, before the development of visible lesions, has been suggested and supported by other findings showing IFN-γ production by initiators of the psoriatic pathogenic cascade, such as autoreactive T cells [146]. Therefore, IFN-γ signaling may likely characterize the early phases of disease, even if not relevantly from the therapeutic point of view, while downstream cytokines, such as IL-17, represent more promising targets. Along these lines: (i) IFN-γ blockade with fontolizumab, an IFN-γ-neutralizing antibody, has shown minimal beneficial effects in treating psoriatic patients, with limited impact on gene expression and modest histological changes [129]; (ii) IL-12 and IFN-γ expression was not reduced when psoriasis was cleared through IL-23 inhibition [147]. 

### 3.3. Interleukin (IL)-17

**Figure 3:**
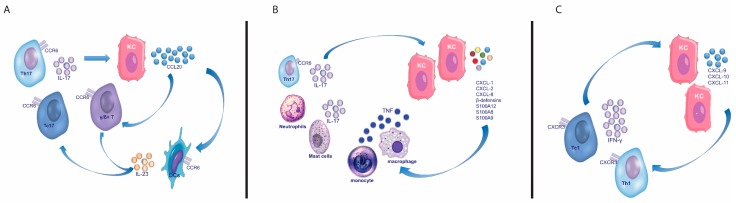
Feed-forward inflammatory circuits involving keratinocytes. IL-17 auto-amplifies its signal through the stimulation of keratinocytes which then produce CCL20 (**A**) or other chemoattractans (**B**) recruiting IL-17-producing T cells (**A**) and other inflammatory cells. In a similar auto-sustaining manner, IFN-γ-secreting T cells are recruited through keratinocyte production of chemokines (CXCL9-11) induced by IFN-γ (**C**). CCL: CC chemokine ligands; CCR: C-C chemokine receptor; CXCL: chemokine (C-X-C motif) ligand; CXCR: C-X-C motif chemokine receptor; IFN: interferon; IL: interleukin; KC: keratinocyte; Th: T helper; Tc: T cytotoxic; TNF: tumor necrosis factor.

IL-17A, generally known as IL-17, belongs to the IL-17 family that includes six members ranging from IL-17 to IL-17F [148]. IL-17 is considered the most relevant cytokine of this class as it shows the highest biological activity and marked inflammatory effects [149]. Increased IL-17 mRNA expression levels and/or protein concentrations have been detected in lesional, uninvolved skin, serum, and tear liquid of psoriatic patients, compared to healthy controls [25,26,27,28,29,30]. This increased expression is associated with a significantly higher number of circulating and skin-infiltrating IL-17+ producing cells [31,42]. IL-17 production is not exclusively dependent on IL-17-producing T cells. In fact, other immune cells, including ILC3, mast cells, and neutrophils, infiltrate lesional skin and contribute to the abundant IL-17 expression [88,95,112,115,118]. IL-17 receptor-bearing tissue cells such as keratinocytes, endothelial cells, and fibroblasts, respond to IL-17 stimulation expressing pro-inflammatory mediators. In particular, keratinocytes respond to IL-17 producing chemokines (CCL20, CXCL-1, -3, -5, CXCL-8, CCL20), AMPs [i.e., LCN2, LL37, DEFB4 (also known as HBD2), S100A proteins], and proinflammatory cytokines, such as IL-6 and IL-1F9 (IL-36γ). Through the production of CCL20, IL-17 drives the recruitment of CCR6+ T cells, which include IL-17+ T cell subtypes (Th17, Tc17, γδ T cells) and mature mDCs [56,85,150] (Figure 3A). Through the induction of CXCL-1, -3, -8 (IL-8) or AMPs, IL-17 sustains neutrophil recruitment, survival, and activation (Figure 3B). In addition, IL-17 can stimulate autoantigen production directly (by inducing KC to produce LL37) or indirectly (by inducing KC to produce CXCL-1, the melanocyte stimulating factor alpha, which induces ADAMSTL5 production by melanocytes). In vitro, IL-17 affects the expression of a large set of genes (more than 600 up- or down-regulated gene probes) in a reconstituted human epidermis model [119], and its effects are amplified by the synergism with other cytokines, including IL-22 and TNF-α, strengthening the production of chemoattractants and AMPs. In lesional psoriatic skin some of these genes are among the most highly expressed genes in the transcriptome and, overall, the in vitro IL-17-regulated gene set is strongly enriched in the psoriasis transcriptome [119]. Although IL-17 mainly exerts proinflammatory effects directly on keratinocytes, it also stimulates keratinocytes to produce IL-19, a cytokine belonging to the IL-20 cytokine family, which shows pro-proliferative effects on keratinocytes themselves [151]. Functional studies showed that IL-17 may induce the psoriasis phenotype, and that its blockade or absence was sufficient to resolve psoriasiform skin lesions in mice models [152,153]. Mechanistic studies on antipsoriatic therapies, such as phototherapy (namely narrow band-UVB, NB-UVB), revealed that their efficacy is strictly correlated to IL-17 signalling suppression, thus demonstrating the advantage of blocking this pathway [137]. This is also true for anti-TNF therapeutics whose efficacy is related to their capability to suppress IL-17, and not TNF-α signalling [154,155]. The final proof of the IL-17 centrality is represented by the striking efficacy obtained by IL-17 antagonists and IL-17 receptor A subunit blocker in reverting clinical, histologic, and molecular features of the psoriasis phenotype in more than 80% of treated patients [11].

### 3.4. Interleukin (IL)-22

IL-22 belongs to the IL-20 cytokine family and it is produced in combination with IL-17, as occurs in Th17, ILC3, and mast cells, or exclusively by specific CD4+ T and CD8+ T cell subsets, named Th22 and Tc22 cells, respectively [42,51,108,156,157]. The expression of the IL-22 receptor is increased in the epidermis of psoriatic lesional skin compared to normal skin, and its effect is mainly directed to keratinocytes. In particular, IL22 (i) enhances keratinocyte migration; (ii) increases epidermal thickness; (iii) inhibits keratinocyte differentiation; (iv) induces the expression of chemokines (i.e., CCL20), neutrophil chemoattractans (i.e., CXCL1, CXCL2, CXCL8), MMPs (i.e., MMP3), platelet-derived growth factor A, AMPs, such as defensin proteins (i.e., DEFβ-2,-3) and S100A protein family (i.e., S100A7, S100A7A, S100A8, S100A9, S100A12), though to lesser extent than IL-17 [90,119,120,121,122,123,124,125,126,155,156,157,158,159,160,161]. IL-22 hyperexpression has been detected in both lesional skin and in the bloodstream, and IL-22 levels correlate with disease severity and significantly decrease during antipsoriatic treatments [27,126,158,161]. Overall, IL-22 in human subjects seems to have weaker pro-inflammatory effects compared to the murine models, wherein IL-22 crucially contributes to the development of a psoriasis-like phenotype and to psoriatic skin inflammation induced by IL-23 or imiquimod. Its blockade or its absence inhibits IL-23- or imiquimod-mediated epidermal hyperplasia in wild-type mice, and it is required to fulfill IL-17 activity during psoriasiform lesion development [152,162]. The pathogenic contribution of IL-22 becomes even more relevant in light of its positive interactions with other cytokine signals. For instance, IL-22 signaling is (i) strengthened by IFN-α that enhances keratinocyte responsiveness via upregulation of IL-22 receptor expression [160]; (ii) its pro-inflammatory activity is potentiated by the synergism with IL-17 and TNF-α; and (iii) the impairing effects on keratinocyte terminal differentiation (including hypogranulosis, parakeratosis, and keratinocyte differentiation gene downregulation) are boosted through keratinocyte-derived IL-20 expression induced by IL-22 itself [125,160]. Nevertheless, IL-22 likely results pathogenically more relevant in animal models of psoriasis and in vitro, rather than in vivo. Notwithstanding a multitude of evidence supporting a central role of IL-22, a modest enhancement of IL-22, compared to IL-17 signaling genes, was detected in the transcriptome of human lesional psoriatic skin [119], and the therapeutic strategy of blocking IL-22 was not successful. Indeed, the development of a IL-22-neutralizing antibody for the treatment of psoriasis, named fezakinumab, was discontinued and switched on atopic dermatitis [130]. 

### 3.5. Interleukin (IL)-23

IL-23 belongs to the IL-6/IL-12 cytokine family. It shows similarities with IL-12 as both are heterodimers constituted by two subunits: the p40 subunit, shared by both cytokines, and p19 or p35 subunit uniquely composing IL-23 or IL-12, respectively [163]. 

Different cell types, including keratinocytes and antigen-presenting cells such as dermal myeloid dendritic cells, macrophages and epidermal Langerhans cells are able to produce IL-23 [164], usually following exposure to bacterial and fungal products binding to TLRs [165]. Moreover, IL-23 expression could be induced by other factors, including TNF-α, IFN-α, TLR ligands, and TSLP [166,167]. IL-23 acts on a wide array of immune cells through the IL-23 receptor complex (IL-23R), expressed on memory T cells, NK cells, neutrophils, mast cells, innate lymphoid cells, and macrophages [168]. Together with TGFβ, IL-1β, and IL-6, IL-23 contributes to the cytokine milieu required for differentiation, expansion, and survival of IL-17-producing T cells [48,169]. Indeed, IL-23 drives the differentiation of CD4+ T cells, CD8+ T cells, γ/δ T cells, and ILC3 in inducing IL-17 expression, but also expression of IL-17F, IL-22, and IL-21 [170]. Additionally, IL-23 stimulates further expression of the IL-23 receptor, thus creating a self-amplificating loop [48]. The centrality of IL-23 is intimately linked to IL-17, which represents the key effector cytokine in its signalling pathway [171,172]. Notably, genome-wide association studies recognized IL-23p19 and IL-23R as susceptibility genes [8,173]. Furthermore, in the psoriatic lesional skin showed an overexpression of IL-12p40 and IL-23p19 compared to non-lesional skin, conversely to IL-12p35 that is not overexpressed [174,175]. The increased expression of IL-23 in lesional psoriatic skin is associated with a marked infiltration of myeloid dendritic cells (CD11c+ dendritic cells), which are the main sources of IL-23 [57]. Consistently, IL-23 serum levels were found significantly higher in psoriatic patients than in healthy controls [176], and expression levels of IL-23 in psoriatic plaques decrease after NB-UVB treatment and biologic therapies, and inversely correlate with clinical responses [137,177,178,179,180]. Functional studies investigating IL-23 contribution to the pathogenesis of psoriasis proved: (i) its ability to induce the development of psoriasiform skin lesions in mice by intradermal injection [181,182,183]; (ii) the inhibition of psoriasis development by injection of IL-23-neutralizing antibodies in two different mice models [153,182]; (iii) the absence of psoriasiform lesions after imiquimod application in IL-23p19 knockout mice in comparison to wild-type mice [62]. Finally, the remarkable efficacy observed in clinical trials testing anti-IL23p19 agents constitutes the confirmatory proof of the IL-23 role in psoriasis [147].

### 3.6. Tumor Necrosis Factor Alpha (TNFα)

TNF-α constitutes a landmark mediator in the pathogenesis of psoriasis since it is the first cytokine to be successfully targeted by therapeutic monoclonal antibodies or fusion proteins for the treatment of the disease. Increased levels of TNF-α have been detected in both lesional skin and serum of psoriatic patients, compared to non-lesional or healthy skin [184,185]. TNF-α is produced by various cell types including T cells, DCs, and keratinocytes [81,82,83,84,85,86,87,88,89]. It shows pro-inflammatory activity that is potentiated by synergistic interactions with other mediators including IL-17 [90,120,121]. It is considered an upstream cytokine in the IL-23/IL-17 pathway, acting as inducer of IL-23 production by DCs [57,154]. 

### 3.7. Anti-Inflammatory and Regulatory Signals Involved in Psoriasis

Regulatory T (Treg) cells represent a subset of T helper cells that limit immune responses and maintain peripheral tolerance, contrasting chronic inflammation, and preventing autoimmune pathogenic process. Their differentiation is driven by a cytokine milieu consisting in TGF-β, IL-4, IFN-γ, IL-2, and IL-6 [186]. Treg cells can be identified by: (i) the high expression of IL-2 receptor alpha chain (CD25); (ii) the expression of transcription factor forkhead box P3 (FoxP3) Foxp3; and (iii) the production of TGF-β, IL-10, perforin, and granzyme A [187,188,189]. Similarly to IL-10-producing Treg cells, other human Treg subsets have been described, such as CD8+ Treg cells and Th3 cells. Treg functional abnormalities and their reduced number have been thought to contribute to psoriatic inflammation, but data are conflicting. However, numerical and/or functional defects within Treg cell subpopulations, likely due to methodological differences or biases related to patient selection, have been reported in psoriasis [187,190]. The imbalance between Treg and effector T cells in the bloodstream of psoriatic patients improved along successful antipsoriatic systemic treatment [191]. In an imiquimod-induced psoriasis mice model, the amelioration of psoriasis-like skin lesions was associated with reduced number of Th17 cytokines and an increased number of Treg cells [191]. On the contrary, at lesional skin level a higher number of Treg cells, compared to control or uninvolved skin, has been detected and their number positively correlated with disease severity. This evidence could suggest a qualitative functional defect of Treg cells in controlling inflammation that is in line with a psoriasis mouse model (knockout for CD18-codifying gene) showing that primary dysfunction of Treg cells determines pathogenic inflammatory T cell proliferation [192]. Furthermore, Treg cells isolated from psoriatic lesional skin or peripheral blood of psoriatic patients demonstrated to be functionally deficient in suppressing effector T cells, upon either alloantigen-specific or polyclonal TCR stimulation [193]. Through the production of IL-10, which downregulates the expression of important proinflammatory cytokines, chemokines, adhesion molecules as well as co-stimulatory molecules, Treg cells could potentially suppress psoriatic inflammation, though clinical trial testing recombinant human IL-10 in psoriatic patients showed modest and transient efficacy [194,195,196]. The anti-inflammatory signal mediated by IL-10 could be potentiated by IL-4 suppressive activity on IL-17 production. Indeed IL-10 stimulates the expression of IL-4 that constitutes a negative regulator of Th17 cell differentiation and keratinocyte activation. Successful antipsoriatic therapies induced IL-4 expression, whose increase is thought to be critical to obtain clinical response [194,195,196]. Notably, recombinant human IL-4 improves psoriasis [197,198,199]. Another functional aspect that needs to be clarified is the pathogenic role of IL-17A-positive, FoxP3-positive Treg cells isolated from lesional skin of psoriasis patients that are oriented towards a pro-inflammatory polarization, loosing FoxP3 expression and increasing levels of RORγt expression levels, similarly to Th17 cells [200].

## 4. The Current Pathogenic Model

Psoriasis can be classified as an IL-23/IL-17-mediated disorder as strongly supported by various lines of evidence. Among them, genetic findings highlighted the importance of IL-23 signaling and the T17 differentiation in psoriasis as some genetic variants of both IL-23 subunits and IL-23R genes confer predisposition to the disease, whereas an IL-23R variant protects against psoriasis [201,202,203,204]. In addition to this axis representing the core of psoriasis pathogenesis, upstream cytokines (IFN-α, IFNγ, and TNFα), synergizing cytokines (IL-22 and TNFα), and downstream mediators (IL-8, IL1F9, and CCL20) complete the pathogenic puzzle (Figure 2B). pDCs, mDCs, and autoreactive T cells, in concert with mast cells and neutrophils, prime the pathogenic cascade. Subsequently, IL-23/IL-17-mediated inflammation, supported by other pro-inflammatory and pro-proliferative molecules derived from T cell activation, induces tissue responses that in turn participate to the pathogenic mechanism, favoring migration of inflammatory cells from bloodstream to the lesional site, proliferation (induction of epidermal hyperplasia and neoangiogenesis), and generation of feed-forward loops that fuel inflammation. This cytokine-driven process is transduced intracellularly by the upregulation of certain signaling pathways, including NF-κB signaling whose initial activation may be genetically determined by CARD14 gene (mapping on PSORS2) variants [205,206]. Similarly, variants of the TRAF3IP2 gene, recognized as another susceptibility gene, affects IL-17 and TNF signaling [207,208,209]. 

### 4.1. Early Phases

**Figure 4:**
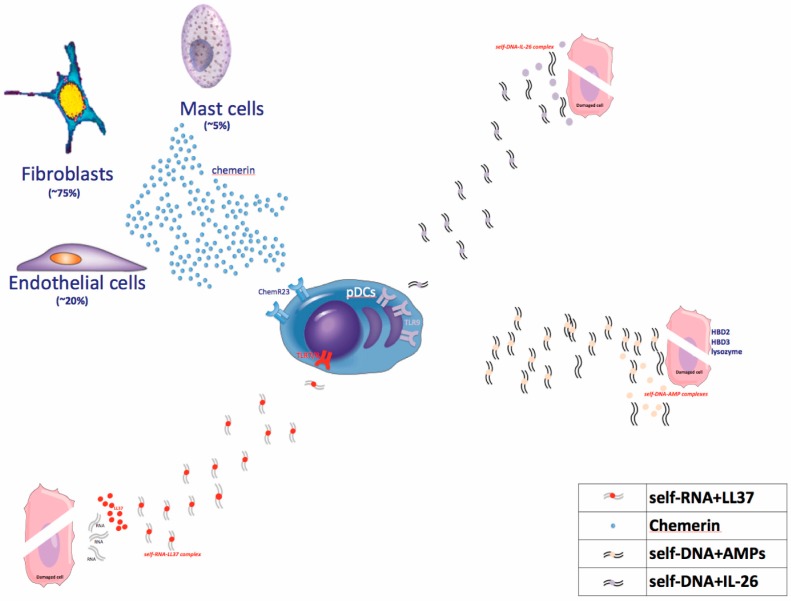
Activation of pDCs. Self-nucleic acids (both DNA and RNA) derived from damaged cells when complexed with AMPs, including LL37, and Th17-derived cytokine IL-26, can activate pDCs through TLR activation. pDCs migration and activation can be also induced by chemerin, an inflammatory protein mainly secreted by fibroblasts. pDCs: plasmacytoid Dendritic Cells; TLR: Toll-like Receptor.

The activation of immune cells, in particular DCs and/or autoreactive T cells, characterizes the early steps of the pathogenic cascade. Because of the immunologic microenvironment, both pDCs and mDCs, once activated, are skewed toward an “inflammatory” phenotype, turning into relevant producers of cytokine and other inflammatory mediators, and becoming mature antigen presenting cells (DC-LAMP+) expressing T cell costimulatory molecules, such as CD86 and HLA-DR. As previously described, pDCs may be activated by various triggers (Figure 4), and represent the initiators of the pathogenic inflammatory cascade through their ability to produce IFN-α. A downstream effect of IFN-α production by pDC is the activation of mDCs, which become highly inflammatory dermal DCs (Tip-DCs), expressing TNF, NO, IL-20, and the p40 cytokines. Within the dermis, IL-23—producing Tip-DCs and mature DC-LAMP+ DCs interact with T cells driving their differentiation towards a dominant IL-17+ T cell phenotype [210]. Another alternative pathway for priming the pathogenic cascade is represented by T cells producing mainly IFN-γ, and to a lesser extent IL-17A. These IL-17—producing lymphocytes are specific for self-antigens, such as LL-37, ADAMSTL-5, and neolipid antigens. Nevertheless, no monoclonal expansion of autoreactive T cells characterizes the T cell compartment as diverse polyclonal α/β and γ/δ T cell repertoires have been detected in lesional psoriatic skin [211]. 

#### 4.1.1. Dendritic Cell Activators

##### TLR Agonists

DCs are activated by diverse TLR agonists, in particular by self-RNA or DNA derived from virus or bacteria. However, self RNA or DNA derived from dying cells may also activate DCs when it is assembled in complexes together with LL37, IL-26, and other AMPs, as they could bind TLR7, -8, or -9. TLR7 and TLR9 are selectively expressed by pDCs, whereas mDCs express TLR3 and TLR8 [212,213,214]. Forming complexes with LL37, self-DNA and self-RNA cannot be degraded and they could bind endosomal TLR7 and -9 in pDCs or TLR8 in mDCs. In particular, self-DNA, when condensed with LL37, DEFB4, hBD3, and lysozyme, is able to trigger pDC activation through TLR9 [70] and to induce IFN-α, while self-RNA complexed with LL37 stimulates mDCs to produce TNF and IL-6 and to become fully mature [72]. Of note, mature DC-LAMP+ mDCs in lesional psoriatic skin co-localize with self-RNA-LL37 complexes [57], and pDCs in lesional psoriatic skin co-localize with LL37 [215]. More recently, a Th17 cytokine with direct antibacterial activity, IL-26, was shown to be highly expressed in psoriasis lesional skin, and to promote pDC-derived IFN-α production when complexed with self-DNA, through TLR9 [73]. 

##### Chemerin

Chemerin is an inflammatory tissue protein produced by fibroblasts, mast cells, and endothelial cells that has been detected in ovarian cancer ascites and in the synovial fluid of rheumatoid arthritis patients [216,217]. Increased levels of chemerin expression has been also detected in lesional psoriatic skin compared to distant uninvolved skin, in atopic dermatitis, and in normal skin. In psoriatic dermis, fibroblasts represent the major source of chemerin which is able to induce pDCs migration in vitro and ERK1/2 phosphorylation [95]. Thus, chemerin, binding to its cognate receptor, chemR23, expressed on pDCs, acts as a chemotactic factor for the recruitment of pDC to prepsoriatic skin [109]. Indeed, chemerin expression specifically marks the early phases of evolving psoriatic skin correlating with pDC migration and activation: chemerin expression patterns are different in chronic stable plaques compared to recent plaques or to unaffected skin adjacent to psoriatic lesions. Along these lines, unaffected adjacent skin, as well as recent lesions, is characterized by strong expression of chemerin in the dermis, accompanied by neutrophil, pDC, and mast cells infiltration [109]. On the contrary, low chemerin expression can be detected in chronic stable plaques showing neutrophil and CD8+ lymphocyte accumulation within the epidermis, but rare pDCs [109,111]. 

##### Thymic Stromal Lymphopoietin (TSLP)

Although TSLP was established as major proallergic cytokine in atopic dermatitis (AD) [218], recently it has been also proved to contribute to human psoriasis physiopathology [166]. TSLP is mainly produced by KCs, while mDCs are the major TSLP-responsive cellular subset in both humans and mice [219,220]. TSLP induces DC maturation and production of inflammatory cytokines (i.e., IL-4, IL-12, and IL-23), that may be synergistically enhanced by CD40L [166,221]. Thus, given the central role of mDC-derived IL-23 in psoriasis, and its relevance in driving IL-17 production, TSLP is becoming a novel player within the complex cytokine network supporting the IL-23/IL-17 axis (Figure 1).

#### 4.1.2. Autoantigens

The identification of the *primum movens* triggering the inflammatory cascade in psoriasis is a fascinating aspect of psoriasis pathogenesis. It has become clear that multiple early triggers could exist, not exclusively linked to DC activation by TLR agonists, as described above.

The presence of autoantigens and autoreactive T cells, and thus an autoreactive mechanism in psoriasis, was suggested by the early 2000s, with the presence of streptococcal M protein-specific T cells cross-reacting against self-antigens (type I keratins). This phenomenon was thought to be due to molecular mimicry induced by the highly similar structure characterizing streptococcal M protein and type I keratins [222,223]. More recently, a psoriasis mice model was developed based on an autoimmune mechanism, wherein injection of IL-17-producing CD4+ T cells recognizing desmoglein 3 as autoantigen was able to develop psoriasis-like lesions [224]. This autoimmune hypothesis has been fostered by the crucial role that the IL-23/IL17 axis plays in other autoimmune disease, and by the strong pathogenic association with HLA-C*06:02, a HLA- Class I molecule, recognized as a psoriasis-susceptibility gene. Nevertheless, the identification of the first autoantigen in psoriatic patients occurred only in the recent years, in 2014, with the detection of circulating and skin-infiltrating autoreactive T cells against LL37 [146], followed by the identification of other autoantigens including ADAMTSL5 and lipid antigens generated by phospholipase A2 group IVD (PLA2G4D) [225,226].

##### LL37

LL37 is secreted by keratinocytes, neutrophils and macrophages, and its expression can be induced by IL-17 stimulation [146]. It is highly expressed in lesional psoriatic skin and it is pathogenically relevant as it forms complexes with extracellular self-nucleic acids activating DCs through TLR7/8/9 [70,71,72]. Its pathogenic relevance has been strengthened by the identification of LL37-specific autoreactive T cells, belonging to both CD4+ and CD8+ T-cell compartments, that were found in 46% of psoriasis patients and even more frequently in moderate-to-severe psoriasis patients [in up to 75% of patients with Psoriasis Activity Severity Index (PASI) > 10] [146]. LL37 is presented by both HLA-Class I (i.e., Cw6*02) and HLA-Class II alleles (HLA-DR1, -DR4, and -DR11,), promoting CD8+ and CD4+ activation, respectively [146]. LL37-targeting T cells secrete key-pathogenic cytokines and chemokines, particularly IFN-γ, but also IL-17, IL-22, IL-21, IL-22, and IL-8, and they express skin-homing chemokine receptors, namely CCR4, CCR6, and CCR10 [146]. 

##### Thrombospondin Type 1 Motif-Like 5 (ADAMTSL5)

A melanocyte-derived protein, ADAMTSL5, has been identified as an autoantigen in 2015 by Prinz’s group [225]. ADAMTSL5 expression is induced by CXCL1, a neutrophil chemoattractant and a melanocyte growth factor, and it is produced by KC upon IL-17 stimulation with IL-17 [225]. ADAMSTL5 expression has been detected not only in melanocytes, but also in keratinocytes throughout the epidermis. The number of melanocytes in psoriatic lesional skin is increased and, notably, T cells, including cytotoxic T cells, co-localize with melanocytes [227]. However, melanocytes detected in psoriatic epidermis do not show signs of cell death, and their number increases in psoriatic lesions, suggesting that melanocytes are likely targets of a non-cytotoxic CD8+ T cell–mediated autoimmune response [224]. Similar to LL37, ADAMTSL5 expression pattern mirrors the infiltrating pattern of T cell and DCs aggregates in the superficial dermis of lesional skin. The high expression of both autoantigen peptides, namely ADAMTSL5 and LL37, in lesional skin co-localizes with DCs, neutrophils, macrophages, and T cells, and it significantly decreases in psoriatic lesional skin treated with either an IL-17 or a TNF blocker [228,229]. This may suggest that ADAMTSL5, as well as LL37, are presented to autoreactive CD4+ T cells by HLA-Class II molecules, and to CD8+ T cells by HLA-Cw6*02, that are expressed on antigen-presenting cell surface within the dermal lymphoid tissue structures [225,229].

##### Lipid Antigens Generated by Phospholipase A2 Group IVD (PLA2G4D)

Besides peptides, lipid-originated antigens may also be recognized as non-self by T cells [226].

Phospholipase A2 group IV (PLA2G4D) is a novel PLA2 enzyme that is absent in normal skin whereas it is highly expressed and shows enhanced activity in psoriatic skin lesions [230]. PLA_2_ expression is detected in mast cells and keratinocytes, and it generates the lipid products that are presented by antigen presenting cells through CD1a, a lipid antigen-presenting protein that shares similarities with HLA-Class I molecules [231]. The interaction between CD1a and PLA2G4D-originating lipid antigens induces activation of T cells and release of IFN-γ, IL-17, and IL-22. CD1a-reactive T cells are increased in the blood and skin of patients affected by psoriasis and they also express CLA+, suggesting their ability to migrate into the skin [232,233].

### 4.2. Amplification Phase and Tissue Cell Response

The amplification phase consists of a wide activation of T cell subsets and other immune cells that boost inflammation and consequent tissue cell responses. In particular, DC activation leads to a reorganization of the dermal T cell infiltration, and to the formation of DCs/T cell clusters that facilitate the activation of the T cell response. Of note, these clusters also co-localize with autoantigens. Moreover, most of the infiltrating dermal DCs secrete IL-23, thus sustaining IL-17-producing T cells. The IL-23/IL-17-driven inflammation is further amplified by the large amounts of pro-inflammatory and pro-proliferative mediators, and it contributes to boost the typical psoriatic tissue cell response, characterized by a typical gene expression profile and histology. In this scenario, keratinocyte-mediated feed-forward circuits are central for inflammatory cell recruitment and for the amplification of inflammatory and proliferative signals. IL-17A acts in synergism with other key-cytokines in psoriasis such as TNF-α and IL-22, stimulating the expression AMPs (LL37, β-defensins, LCN2, S110A family proteins), inflammatory cytokines (IL-1 family members and IL-6), and chemokines (CXL1, -3, -5, -8, and CCL20). In particular, IL-17, together with IL-22 and TNF-α, stimulates KCs to produce CXCL-1, -3, and -8, chemokines attracting neutrophils and sustaining their activation and survival. This synergism increases also the production of CCL20, important for recruitment of CCR6+ skin-homing cells, such as IL-17-producing T cells, IL-22-producing T cells, and DCs [54,86,87,119]. Additionally, the CCL20/CCR6 chemokine system, together with the CCL19/CCR7 axis, is centrally involved in the dermal lymphoid aggregate formation. These aggregates consist of mature mDCs expressing DC-LAMP/CD208, CD11c, HLA-DR, CCR6, and T cells expressing CCR6+ that produce both IL-17 and CCL20 [86,87,234,235]. The formation of these clusters induces the in situ activation of T cells, and it represents the downstream effect of DC activation and maturation by autoantigens or TLR ligands [236,237]. The massive presence of mature DC-LAMP+ DCs aggregated with T cells contributes to the chronic inflammatory process and it correlates with clinical induration scores of psoriatic plaques and with disease severity. Moreover, IL-17 could drive broad feed-forward loops enhancing directly or indirectly the expression of far upstream mediators such as LL37 and CXCL1. On the other hand, TLRs, which are considered crucial in the early steps of the pathogenesis of the disease, could also have effects on far downstream steps once inflammation is established. In fact, the inhibition of TLR7, 8, and 9 is able to suppress IL-23-induced inflammation in a mice model, decreasing also the IL-17 signature genes and the down-stream IL-17 signaling [238].

All together these results support the concept that in psoriasis a vicious loop reverberates the IL-17 signal within the lesional site. 

In a similar manner, IFN-γ amplifies IFN-γ signaling and induces the recruitment of IFN-γ-producing cells, via KC production of CXCL9, CXCL10, and CXCL11, that attract CXCR3+ T cells which are highly enriched in IFN-γ-producing T cells (Figure 3C) [89]. Though KCs are considered the key responding cells to the cytokine microenvironment, the contribution of other tissue cells should be underscored. The relevance of melanocytes in the pathogenesis of the disease has become more appreciated since the identification of a melanocyte-derived autoantigen. Endothelial cells favor inflammatory cell migration into lesional sites through the expression of adhesion molecules including ICAM-1, VCAM-1, ELAM-1, HECA-452, and 4D10I-CAM [239,240]. Fibroblasts also secrete chemerin, other pro-inflammatory products such as IL-6, and MMPs [119].

## 5. The Pathogenic Cascade Compendium

The early steps of the pathogenic cascade consists in the activation of IFN-α-producing pDCs triggered by TLR agonists, and/or the activation of autoreactive T cells producing IFN-γ, and other key-cytokines such as IL-17. While autoreactive T cells may potentially initiate the pathogenic process, leading to the psoriatic phenotype, pDCs, through their IFN-α-production, stimulate mDC to become highly inflammatory dermal DCs producing TNF-α, IL-23, IL-20, and NO. Besides IFN-α, they may be also stimulated by TSLP and TNF-α. Their IL-23 production stimulates IL-17 producing cells, which include Th17, Tc17, γδ T cells, ILC3, mast cells, and neutrophils. IL-17, in cooperation with other cytokines such as TNFα and IL-22, induces the development of the psoriasis phenotype through tissue cell activation. The most relevant tissue response is provided by keratinocytes that, releasing chemokines and other pro-inflammatory molecules (AMPs), sustain skin inflammation.

## 6. Conclusions

Nowadays, psoriasis is the best-studied immune-mediated skin disease. Multiple cytokine-mediated signaling pathways can be traced within the psoriasis transcriptome, although just a few turned out to be crucial for the development of the psoriasis phenotype, with their blockade being therapeutically advantageous. The classic view of psoriasis pathogenesis was established on the IL-12/Th1 pathway but has now been profoundly revised, and “under the IL-17 light”, the pathogenic role of IFN-γ has been reconsidered, placing it in the early steps of the psoriatic cascade, and acting as an activating factor for antigen-presenting cells. The current pathogenic model is centered on the IL-23/IL-17 axis, and it is being dynamically integrated and remodeled according to new acquisitions, such as the recent identification of autoantigens and autoreactive T cells. However, many aspects still need to be elucidated, and their clarification will help to better understand the pathology of psoriasis and to improve the therapeutic strategy against this disease.

## Abbreviations

β-DEF	β-defenin
CCL	CC chemokine ligands
CXCL	chemokine (C-X-C motif) ligand
CLA	cutaneous lymphocyte antigen
DCs	dendritic cells
EGF	epidermal growth factor
ELAM-1	endothelial leukocyte adhesion molecule-1
GWAS	genetic-wide association studies
HBEGF	heparin-binding EGF-like growth factor
HLA	Human leukocyte antigen
ICAM-1	intercellular adhesion molecule-1
IFN	interferon
IL	interleukin
ILC	innate lymphoid cells
iNOS	intracellular nitric oxide synthase
KCs	keratinocytes
mDC	myeloid Dendritic Cell
MMPs	matrix metalloproteinases
NKT	natural Killer T cell
NO	nitric oxide
pDC	plasmacytoid Dendritic Cells
STAT	signal transducer and activator of transcription
TLR	toll-like receptor
TNF	tumor necrosis factor
TSLP	thymic stromal lymphopoietin
VCAM-1	vascular cell adhesion protein 1
VEGF	vascular endothelial growth factor
